# Parental Awareness of Headaches Among Elementary School-Aged Children in Makkah, Saudi Arabia: A Cross-Sectional Study

**DOI:** 10.7759/cureus.44331

**Published:** 2023-08-29

**Authors:** Mohammad K Dhafar, Faris Y Bahakeem, Anas H Alsehli, Rawan S Kofyah, Ruba E Hamad, Weaam I Faraj, Bayader S Alsalem, Mohamed A Elhefny

**Affiliations:** 1 Department of Medicine and Surgery, College of Medicine, Umm Al-Qura University, Makkah, SAU; 2 Department of Genetics, College of Medicine, Umm Al-Qura University, Makkah, SAU

**Keywords:** health education & awareness, makkah, pediatrics, children, school, parents, headache

## Abstract

Background

Headaches are a prevalent form of pain experienced in the skull, face, and facial structures, categorized into two types: primary and secondary. Primary headaches are more frequently observed in children and can be triggered by sleep disturbances, vision problems, malnutrition, and other medical conditions. Despite their prevalence among children, these headaches often go unrecognized and untreated, and there seems to be insufficient parental awareness regarding childhood headaches. This study aims to determine the prevalence of childhood headaches and assess awareness about this among parents of elementary school-aged children in Makkah, Saudi Arabia, to enhance parental understanding of this issue.

Methods

Data were collected through an online survey created using Google Forms (Google LLC, Mountain View, California, United States), distributed to parents residing in Makkah. The survey was disseminated in March 2023.

Results

A total of 499 parents completed the study questionnaire, comprising 399 mothers and 100 fathers, with a mean parental age of 37.1 ± 13.9 years. Of the participants, 89.2% were married, 91% were Saudi nationals, and 105 had secondary education. Parents reported that 13.2% of their children complained of headaches, with 55.3% describing them as occasional. Among the respondents, 178 parents sought medical care for their children’s headaches primarily out of fear. It was found that 69.7% of parents with higher education had good knowledge regarding childhood headaches, while 59.8% of employed parents had good knowledge compared to 43.8% of others.

Conclusion

This study revealed a lack of adequate knowledge and awareness among parents regarding headaches in children residing in Makkah. Therefore, we recommend conducting further research and implementing educational initiatives to enhance parental understanding of childhood headaches in Makkah and other regions of Saudi Arabia.

## Introduction

A headache is characterized by pain experienced above the orbitomeatal line, encompassing the skull, face, and facial structures. There are two types of headaches: primary and secondary [1,2]. Primary headaches, more prevalent in children, are not associated with an underlying disease and include migraines, tension headaches, cluster headaches, and trigeminal autonomic cephalgias. Secondary headaches, on the other hand, are caused by pre-existing conditions such as head trauma and meningitis [1,3]. Sleep disturbances, vision problems, malnutrition, dental issues, and other medical conditions have been identified as potential triggers for headaches.

Headaches are a common reason for seeking medical consultation in both adult and pediatric neurology clinics [4]. They have a substantial impact on children and their families, particularly primary headaches, which adversely affect social functioning, school performance, and overall quality of life [5]. Sixty percent of children report having at least three headache attacks each year. Headaches often remain unrecognized and undertreated. Only a small fraction of children with challenging headache disorders are referred to and managed at specialized pediatric facilities, while most are managed at home with over-the-counter drugs or receive care in primary settings [6].

A recent study conducted in Riyadh, Saudi Arabia in 2021, revealed that 62.3% of the parents reported their children complaining of headaches; the use of electronic devices was incriminated in about 47.6% and overall parents' awareness was about 55.1% [1]. In another study by Al Jumah et al., the observed one-year prevalence of all headaches was 77.2%; after adjustment, it dropped to 65.8%. Adjusted one-year prevalence for headache types was as follows: migraine 25.0% and tension-type headache (TTH) 34.1% [7]. Approximately 42% of young people with headaches face limitations in everyday life, and practically all children and adolescents with headaches experience a decline in health-related quality of life [8]. Improving awareness regarding early diagnosis and preventive therapies is crucial to prevent chronic headaches and mitigate their negative impact on school performance and social interactions. Children's and young adults' headache disorders place a financial strain on families and society. Children and their families are negatively impacted by pediatric headaches. Primary headache diseases also impair quality of life and negatively affect social and academic activities. In a study by Al-Hashel et al. in 2019, the one-year prevalence of primary headache was 19.4% in the age group of 6-17 years; it was 10.4% for children aged 6-11 years and 25.8% for those aged 12-17 years [5].

To the best of our current understanding, there is a lack of research examining the level of parental awareness regarding headaches in elementary school-aged children residing in the western region of Saudi Arabia, specifically in Makkah. The distinctive cultural, environmental, and demographic elements present in Makkah may potentially yield divergent outcomes in contrast to those documented elsewhere [9,10]. For example, the distinct climate, air quality, and lifestyle factors in Makkah may have an impact on the frequency of headaches and the level of parental awareness within this particular population [11,12]. Furthermore, there is a lack of research investigating the frequency of headache etiology and associated factors, including birth order, gender, and family size, within this particular demographic [13].

This study aims to evaluate the occurrence of headaches in children and the level of parental knowledge regarding headaches among elementary school students in Makkah, Saudi Arabia, in 2023. The study aims to specifically identify the prevailing causes of headaches among the population of Makkah, evaluate the extent of parental awareness regarding this issue, and examine the potential correlation between parental awareness and the prevalence of headaches. The primary objective of this study is to augment parental knowledge regarding headaches in the targeted age cohort, while also making a valuable contribution to the existing body of scholarly literature on this subject matter.

## Materials and methods

This study was performed by researchers from the University of Al-Qura (UQU). UQU is a public university located in Makkah, Saudi Arabia. The study was approved by the Biomedical Ethics Committee of UQU (approval number: HAPO-02-K-012-2023-02-1448). The principal investigator ensured strict confidentiality of all study participants, with no personal identifiers used in the final manuscript. The primary objectives of the study were to assess the prevalence of headaches as well as parental awareness of headaches among elementary school-going children in Makkah. Parental knowledge was assessed in terms of the clinical signs, symptoms, and types of headaches that their children can encounter. The demographic and socio-economic factors associated with parental knowledge of childhood headaches were also identified.

This cross-sectional study was conducted in 2023 among parents of children enrolled in public and private elementary schools in Makkah. Parents were recruited for this study through social media platforms, specifically WhatsApp (Meta Platforms, Inc., Menlo Park, California, United States) and Telegram (Telegram Group Inc., Tortola, British Virgin Islands). The study investigators provided counseling to parents, explaining the purpose and methodology of the research. They assured the participants that their identities and personal information, as well as those of their children, would be protected and kept confidential. To confirm their participation, electronic informed consent was obtained from the parents. As the forms were distributed online, hand-written signatures could not be obtained for consent. However, the parents gave consent online prior to answering the questionnaire.

Parents of elementary school-going children in Makkah, with children of age between 6 to 12 years, who provided consent to participate were included in this study. Parents of older children attending high school, aged 12 years and above, were excluded. A convenience sampling technique was employed. The required sample size for the study was determined using OpenEpi website (Open Source Epidemiologic Statistics for Public Health, Version 3.0), taking into account the following parameters: the population size of Makkah city (estimated to be around 2 million people), a confidence interval (CI) level of 95%; and assuming a 50% prevalence of headaches. Based on these factors, a total of 384 participants were calculated to be the required sample size. The prevalence of headaches was set at 50%, based on the findings of a previous study conducted in 2019 among 1400 school-going children in Riyadh, which reported an overall headache prevalence of 49.8% in their study population [4].

Data collection was conducted in March 2023 using an online, self-administered questionnaire. The required sample size was achieved successfully within one month of starting data collection. The questionnaire was created using Google Forms (Google LLC, Mountain View, California, United States) and was adapted from a similar study conducted in Riyadh, with permission obtained from the corresponding author [1]. As this questionnaire has already been used in the aforementioned previous study, we did not carry out a pilot study to validate it further. The questionnaire, available in Arabic language, was distributed to parents through social media platforms WhatsApp and Telegram.

The questionnaire was divided into three distinct sections, each focusing on specific components: Section 1: research objectives and a consent form, Section 2: demographic information of parents and their children, and Section 3: questions to assess parental awareness of childhood headaches.

The data was analyzed using IBM SPSS Statistics for Windows, Version 26.0 (Released 2019; IBM Corp., Armonk, New York, United States). The Shapiro-Wilk test was used to identify whether quantitative variables were normally distributed or skewed. For normally distributed quantitative variables, specifically parental age, the mean and standard deviation were calculated. Median and interquartile range (IQR) were reported for quantitative variables with a skewed distribution, namely parental income. Frequencies and percentages were reported for demographic and socio-economic factors such as gender, nationality, education, and employment status. The Pearson Chi-square test was used to identify factors associated with parental knowledge of childhood headaches. A p-value of less than 0.05 was considered statistically significant for all data analyses.

## Results

A total of 499 parents completed the study questionnaire, with 399 (80%) being mothers and 100 (20%) being fathers. The age of respondents ranged from 18 to over 45 years, with a mean age of 37.1 ± 13.9 years. Of the total, 445 (89.2%) parents were married, and 454 (91%) were Saudi. Regarding educational level, 105 (21%) had secondary education or below, while 328 (65.7%) had a bachelor’s degree or diploma. Among the participants, 259 (51.9%) were employed, and 68 (13.6%) worked in the healthcare field. In terms of monthly income, 254 (50.9%) reported a monthly income less than 10,000 SR, 208 (41.7%) reported a monthly income of 10,000-20,000 SR, and 37 (7.4%) reported an income of more than 20000 SR. A total of 103 (20.6%) were smokers, and 43 (8.6%) were infrequent smokers (Table 1).

**Table 1:** Parents' demographic data, Makkah, Saudi Arabia SR: Saudi Riyal

Parents demographic data	Number	Percentge
Respondent		
Father	100	20.0%
Mother	399	80.0%
Respondent's age		
18-25 years	28	5.6%
26-35 years	181	36.3%
36-45 years	198	39.7%
> 45 years	92	18.4%
Marital status		
Married	445	89.2%
Divorced/widowed	54	10.8%
Nationality		
Saudi	454	91.0%
Non-Saudi	45	9.0%
Educational level		
Secondary or below	105	21.0%
Bachelor's degree/diploma	328	65.7%
Postgraduate degree	66	13.2%
Employment		
Yes	259	51.9%
No	240	48.1%
Working in healthcare field		
Yes	68	13.6%
No	431	86.4%
Monthly income		
< 10000 SR	254	50.9%
10000-20000 SR	208	41.7%
> 20000 SR	37	7.4%
Smoking		
Yes	103	20.6%
Sometimes	43	8.6%
No	353	70.7%

Children’s ages ranged from six to 12 years, with a mean age of 9.1 ± 2.0 years old. A total of 248 respondents (49.7%) had two or fewer children, 187 (37.5%) had three to four children, and 64 (12.8%) had five or more children. A total of 203 children (40.7%) were the first child, and 155 (31.1%) were the last child. A total of 274 children (54.9%) were males (Table 2).

**Table 2:** Demographic characteristics of children of the study participants in Makkah, Saudi Arabia

Children's data	Number	Percentage
Age of child		
6-7 years	128	25.7%
8-9 years	145	29.1%
10-12 years	226	45.3%
Number of siblings		
2 or less	248	49.7%
3-4	187	37.5%
5 or more	64	12.8%
Child's order		
First	203	40.7%
Middle	141	28.3%
Last	155	31.1%
Child's gender		
Male	274	54.9%
Female	225	45.1%

As for susceptibility, 74.3% agreed that headache affects people of all ages from childhood to old age, 66.7% reported that children could have TTH, 41.7% agreed that children could have migraines, and 36.1% agreed that children could have cluster headaches. Considering causes of headaches, 91.2% knew that childhood headaches could be caused by sleep disturbances, 90% knew that childhood headaches could be due to vision problems, 86.4% agreed that childhood headaches could be due to malnutrition, 85.8% reported that childhood headaches could be due to dental problems, and 84.8% know that childhood headaches can be secondary to other medical conditions. Concerning headache symptoms, 77.6% reported dizziness, 75.8% reported blurred vision, 72.3% reported fever, 58.7% were told for vomiting, and 51.9% for speaking difficulty or altered consciousness (Table 3).

**Table 3:** Parents' knowledge regarding headaches among children in Makkah, Saudi Arabia (Part 1)

Knowledge items	Strongly disagree		Disagree		Neutral		Agree		Strongly agree	
	No	%	No	%	No	%	No	%	No	%
Susceptibility										
Headache affects people of all ages from childhood to old age	3	.6%	25	5.0%	100	20.0%	196	39.3%	175	35.1%
Children can have migraines	9	1.8%	117	23.4%	165	33.1%	128	25.7%	80	16.0%
Children can have tension-type headache	8	1.6%	45	9.0%	113	22.6%	207	41.5%	126	25.3%
Children can have cluster headache	17	3.4%	108	21.6%	194	38.9%	115	23.0%	65	13.0%
Causes of headache										
Childhood headaches can be secondary to other medical conditions	3	.6%	12	2.4%	61	12.2%	241	48.3%	182	36.5%
Childhood headaches can be due to psychological causes	6	1.2%	30	6.0%	83	16.6%	231	46.3%	149	29.9%
Childhood headaches can be due to malnutrition	3	.6%	17	3.4%	48	9.6%	225	45.1%	206	41.3%
Childhood headaches can be due to sleep disturbances	3	.6%	10	2.0%	31	6.2%	221	44.3%	234	46.9%
Childhood headaches can be due to vision problems	4	.8%	15	3.0%	31	6.2%	187	37.5%	262	52.5%
Childhood headaches can be due to dental problems	5	1.0%	17	3.4%	49	9.8%	195	39.1%	233	46.7%
Childhood headaches can be due to a brain tumor or hemorrhage	7	1.4%	30	6.0%	76	15.2%	157	31.5%	229	45.9%
Symptoms of headache										
Childhood headaches can be accompanied by vomiting	9	1.8%	57	11.4%	140	28.1%	181	36.3%	112	22.4%
Childhood headaches can be accompanied by speaking difficulty or altered consciousness	6	1.2%	83	16.6%	151	30.3%	162	32.5%	97	19.4%
Childhood headaches can be accompanied by dizziness	3	.6%	25	5.0%	84	16.8%	234	46.9%	153	30.7%
Childhood headaches can be accompanied by blurred vision	5	1.0%	25	5.0%	91	18.2%	214	42.9%	164	32.9%
Childhood headaches can be accompanied by fever	5	1.0%	39	7.8%	94	18.8%	204	40.9%	157	31.5%

Most (65.7%) of the parents reported that they would go to an emergency clinic if a child gets a headache after falling on his/her head, 53.5% will go if the child has a headache and vomiting, 49.9% go to an emergency room (ER) if the child has changes in consciousness along with headache, and 38.1% go to ER if the child develops fever along with headache (Table 4).

**Table 4:** Parents' knowledge regarding headache among children in Makkah, Saudi Arabia (Part 2)

Management of headache	Go to emergency clinic		Book a normal appointment		Let the child sleep		Wait until the next day		Give medication from personal expertise		Doesn’t seem that serious	
	No	%	No	%	No	%	No	%	No	%	No	%
If your child is crying from the severity of headache	125	25.1%	36	7.2%	96	19.2%	38	7.6%	189	37.9%	15	3.0%
If your child has changes in consciousness along with headache	249	49.9%	104	20.8%	45	9.0%	56	11.2%	29	5.8%	16	3.2%
If your child develops fever along with headache	190	38.1%	64	12.8%	27	5.4%	24	4.8%	180	36.1%	14	2.8%
If your child has headache and vomiting	267	53.5%	53	10.6%	24	4.8%	29	5.8%	110	22.0%	16	3.2%
If your child has headache for 6 months	253	50.7%	151	30.3%	19	3.8%	26	5.2%	37	7.4%	13	2.6%
If your child gets headache after falling on his/her head	328	65.7%	47	9.4%	27	5.4%	37	7.4%	44	8.8%	16	3.2%

A total of 13.2% of the parents reported that their children complained of headaches, and 55.3% said they complained sometimes. An exact 20.2% said that a child’s headache prevents the child from daily activities, and 36.3% said that a headache somehow affects the child’s day/performance. A total of 178 (52%) of the parents sought medical care for headaches in their children mainly due to fear (47.2%), concerns about activities of daily living (25.3%), concerns about vision problems (18.5%), and concerns about seizures (9%). The most reported diagnosis was headache due to multifactorial causes (27%), use of electronics (23.6%), other medical causes (23.6%), vision problems (18.5%), and sleep deprivation (2.2%) (Table 5).

**Table 5:** Parents' feedback on their child's headache history and their practice

Parents practice	Number	Percentage
Does your child complain of headaches?		
Yes	66	13.2%
Sometimes	276	55.3%
No	157	31.5%
Severity of the headaches		
Unremarkable/negligible effect	149	43.6%
Somehow affects the child’s day/performance	124	36.3%
Prevents the child from daily activities	69	20.2%
Parents sought medical care for headaches in their children		
Yes	178	52.0%
No	164	48.0%
Motive for seeking medical care		
Fear	84	47.2%
Concerns about activities of daily living	45	25.3%
Concerns about vision problems	33	18.5%
Concerns about seizures	16	9.0%
Physician diagnosis		
Multifactorial	48	27.0%
Use of electronics	42	23.6%
Other medical causes	42	23.6%
Vision problems	33	18.5%
Other causes	9	5.1%
Sleep deprivation	4	2.2%

**Figure 1:**
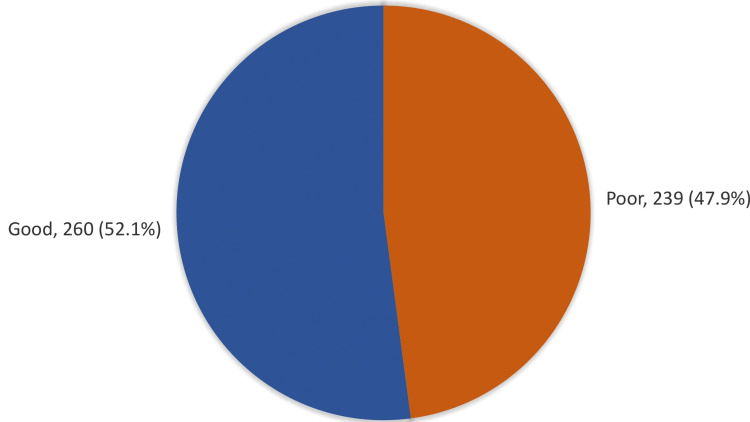
Overall parental knowledge level regarding headache among children in Makkah, Saudi Arabia

Of the parents who participated in the study, 260 (52.1%) had a good overall knowledge level, and 239 (47.9%) had a poor overall knowledge level (Figure 1).

Most (69.7%) parents with post-graduate degrees had good knowledge levels versus 40% of others with lower education; this difference was statistically significant (p = 0.001). Also, 59.8% of employed parents had good knowledge compared to 43.8% of others (p = 0.001). Good knowledge was detected among 66.2% of those in the healthcare field versus 49.9% of those in other fields (p = 0.012). Also, 57.3% of those with two siblings or less had good knowledge compared to 42.2% of those with five or more children (p = 0.049) (Table 6).

**Table 6:** Factors associated with parent's knowledge regarding headache among children, Makkah, Saudi Arabia * P < 0.05 (significant) SR: Saudi Riyal

Factors		Overall knowledge level				p-value
		Poor		Good		
		No	%	No	%	
Respondent	Father	41	41.0%	59	59.0%	.123
	Mother	198	49.6%	201	50.4%	
Respondents age in years	16-25	11	39.3%	17	60.7%	.072
	26-35	75	41.4%	106	58.6%	
	36-45	102	51.5%	96	48.5%	
	> 45	51	55.4%	41	44.6%	
Social status	Married	212	47.6%	233	52.4%	.743
	Divorced / widow	27	50.0%	27	50.0%	
Nationality	Saudi	213	46.9%	241	53.1%	.164
	Non-Saudi	26	57.8%	19	42.2%	
Educational level	Secondary / below	63	60.0%	42	40.0%	.001*
	Bachelor / diploma	156	47.6%	172	52.4%	
	Post-graduate	20	30.3%	46	69.7%	
Employment	Yes	104	40.2%	155	59.8%	.001*
	No	135	56.3%	105	43.8%	
Work at health care field	Yes	23	33.8%	45	66.2%	.012*
	No	216	50.1%	215	49.9%	
Monthly income	< 10000 SR	129	50.8%	125	49.2%	.108
	10000-20000 SR	98	47.1%	110	52.9%	
	> 20000 SR	12	32.4%	25	67.6%	
Child age in years	6-7	58	45.3%	70	54.7%	.020*
	8-9	58	40.0%	87	60.0%	
	10-12	123	54.4%	103	45.6%	
Number of siblings	2 or less	106	42.7%	142	57.3%	.049*
	3-4	96	51.3%	91	48.7%	
	5 or more	37	57.8%	27	42.2%	
Child order	First	89	43.8%	114	56.2%	.235
	Middle	68	48.2%	73	51.8%	
	Last	82	52.9%	73	47.1%	
Child gender	Male	133	48.5%	141	51.5%	.751
	Female	106	47.1%	119	52.9%	

## Discussion

The primary objective of this study was to evaluate parental awareness concerning headaches among primary school-aged children in Makkah. A total of 499 parents participated by completing the study questionnaire, with the majority being mothers (80%). The respondents’ mean age was 37.1 ± 13.9 years, ranging from 18 to over 45 years. This research included parents with children below the age of 12, in contrast to Yousef et al.’s study in which the sample encompassed children aged 14 and above [14].

The educational level of the respondents revealed that 65.7% possessed at least a bachelor’s degree or diploma, whereas only 21% completed only secondary education or below that level. This observation aligns with the prevailing pattern in Saudi Arabia, where there has been a growing focus on education, particularly for women [15]. This trend can also be attributed to the study’s location in primary healthcare centers, which are commonly situated in urban areas with higher educational attainment

Regarding employment status, over half of the respondents (51.9%) were employed, with 13.6% working in the healthcare sector. Comparable findings were observed in a study conducted in Madinah, which reported a significant proportion of employed parents seeking care at primary healthcare centers [16]. With regards to monthly income, approximately half of the participants (50.9%) reported earnings below 10,000 Saudi Riyal (SR), while 41.7% indicated a monthly income of 10,000-20,000 SR. A smaller percentage, constituting 7.4%, reported an income exceeding 20,000 SR.

Concerning susceptibility, most parents (74.3%) agreed that headaches affect individuals of all ages, ranging from childhood to old age. This finding is consistent with prior research that has established headaches as a prevalent issue across all age groups, including children [17]. Headaches can occur in individuals of all ages, including children, due to various factors such as stress, inadequate sleep patterns, dehydration, eye strain, or infections. In children, headaches can also arise from medical conditions such as migraines or tension headaches. Furthermore, 66.7% of parents reported that children could experience TTH, which is the most commonly observed headache type in children.

The study additionally revealed that 41.7% of parents acknowledged that children could experience migraines, representing a lower percentage than previous studies’ findings. For example, Al Khalili et al. reported that parents demonstrated awareness of migraines in children [18]. This disparity may be attributed to cultural and regional discrepancies in parental awareness and perceptions regarding headaches.

Moreover, the study indicated that merely 36.1% of parents agreed that children could experience cluster headaches. This finding aligns with the rarity of cluster headaches among children [19]. Cluster headaches are infrequent in this age group as they are predominantly associated with older individuals and more prevalent in adults. Cluster headaches are believed to involve hypothalamus dysfunction, which might manifest differently in children than adults. Nonetheless, the limited awareness among parents could potentially lead to misdiagnosis and delayed treatment for children experiencing cluster headaches.

Concerning the causes of headaches, the study revealed that the majority of parents were aware that childhood headaches could be attributed to sleep disturbances (91.2%), vision problems (90%), malnutrition (86.4%), dental problems (85.8%), and other medical conditions (84.8%). These findings align with studies conducted in 2007, which identified these factors as prevalent causes of headaches in children [20].

Our study also evaluated parents’ awareness of headache symptoms in children. The majority of parents identified dizziness (77.6%), blurred vision (75.8%), fever (72.3%), vomiting (58.7%), and speech difficulties or altered consciousness (51.9%) as common symptoms associated with headaches in children. These findings align with a study by Paschou et al., which recognized these symptoms as frequently observed in children experiencing headaches [21].

According to the data presented in Table 4, most parents (65.7%) indicated they would take their child to the emergency clinic if the child experienced a headache after a head injury. This finding aligns with the National Institute for Health and Care Excellence clinical guidelines for head injury management, which advise seeking medical attention for any head injury accompanied by persistent headache, loss of consciousness, or other worrisome symptoms [22].

Furthermore, more than half of the parents (53.5%) indicated that they would take their child to the emergency clinic if they experienced both a headache and vomiting. This combination of symptoms can indicate various serious conditions, including meningitis or a brain tumor, emphasizing the importance of immediate medical assessment to ensure accurate diagnosis and appropriate treatment [23].

Nearly half of the parents (49.9%) stated that they would seek emergency medical attention if their child exhibited changes in consciousness alongside a headache. This response reflects a commendable level of awareness concerning the potential gravity of altered mental status, which can signify a significant underlying condition [24].

A smaller subset of parents (38.1%) indicated they would bring their child to the emergency clinic if the child experienced a fever and a headache. Although fever can be a common symptom of various viral illnesses, it is generally not a cause for significant concern when experienced alone. However, if accompanied by other worrisome symptoms like a headache, it may warrant prompt medical evaluation [25]

Based on the findings of our study, it was observed that 13.2% of parents reported their children complaining of headaches, with 55.3% indicating occasional occurrence. Additionally, 20.2% of parents believed that their child’s headaches hindered their daily activities, while 36.3% perceived some impact on their child’s day-to-day performance. Among the surveyed parents, 52% sought medical care for their child’s headaches, primarily driven by fear (47.2%), concerns about daily activities (25.3%), vision problems (18.5%), and apprehensions about seizures (9%). The most frequently reported diagnosis was headaches due to multifactorial causes (27%), followed by electronic device usage (23.6%), other medical causes (23.6%), vision problems (18.5%), and sleep deprivation (2.2%).

Regarding parents’ overall knowledge level regarding headaches in children, 52.1% demonstrated good knowledge, while 47.9% exhibited poor knowledge. This indicates the existence of room for improvement in educating parents about the causes, symptoms, and treatment of headaches in children. These findings align with prior research highlighting the high prevalence of headaches in children and the necessity for enhanced awareness among parents and healthcare professionals [8]. Moreover, a study conducted in Malaysia discovered that parental knowledge regarding the causes and management of headaches was generally deficient, with many parents resorting to traditional remedies instead of seeking medical attention [26].

Our study illustrates a significant association between parents’ level of education and their knowledge regarding headaches in children. Parents with post-graduate degrees exhibited a good level of knowledge at 69.7%, compared to 40% of parents with lower education levels (p = 0.001). This finding aligns with a previous study conducted in 2016, which demonstrated a positive correlation between education level and health knowledge [27]. This association can be attributed to the fact that education equips individuals with the knowledge and skills required to access and comprehend health-related information.

Furthermore, the study revealed a significant association between employment status and parents’ knowledge regarding headaches in children. Employed parents exhibited a good knowledge level of 59.8%, compared to 43.8% among non-employed parents (p = 0.001). This finding could be attributed to the fact that employed parents often have access to more resources, including health insurance and paid time off, which enable them to seek medical advice and stay informed about current health information [1].

Moreover, the study revealed a significant disparity in knowledge levels regarding headaches in children between parents working in the healthcare field and those in other fields. Among healthcare workers, a good knowledge level was observed in 66.2% of participants, while the percentage was 49.9% for non-healthcare workers (p = 0.012). This discrepancy can be attributed to healthcare workers having greater access to pertinent and current medical information and increased exposure to patients with headaches, which enhances their knowledge and awareness of the condition [28].

Lastly, the study identified a significant association between a child’s number of siblings and parents’ knowledge regarding headaches in children. Parents with two or fewer children demonstrated a good knowledge level of 57.3%, whereas the percentage was 42.2% among parents with five or more children (p = 0.049). This finding may be attributed to the notion that parents with fewer children often possess more time and resources to dedicate to each child’s health and well-being, including seeking information about headaches and other health-related matters [29]. With fewer children to care for, parents might be able to allocate more attention to each child’s specific needs, such as monitoring their health and promptly seeking medical assistance when necessary.

Limitations

Limitations of this study include the exclusive focus on the Makkah population, which may limit the generalizability of findings to other regions. Additionally, the reliance on online questionnaires restricted participation to individuals with internet access, potentially introducing bias. Recall bias could also affect the accuracy of responses. Furthermore, the study did not explore the potential impact of cultural, socioeconomic, or linguistic factors on parental awareness. Lastly, the convenience sampling technique employed might not fully represent the diverse population of elementary school-aged children and their parents. Further research could benefit from addressing these limitations to provide a more comprehensive understanding of parental awareness of childhood headaches in Saudi Arabia.

## Conclusions

This study highlights the inadequate knowledge of parents regarding headaches among children in Makkah. Therefore, it is imperative to educate parents through public campaigns, social media platforms, and e-brochures, disseminating information about headaches, their causes, associated symptoms, and appropriate management strategies. Additionally, we recommend conducting further studies in different regions of Saudi Arabia to assess parental knowledge regarding headaches among children across the kingdom.
